# Hematologic and biochemical reference values for anesthetized juvenile German crossbred farm pigs

**DOI:** 10.1038/s41598-024-78317-2

**Published:** 2024-11-05

**Authors:** Florian Meissner, Johannes Dinkelaker, Alexander Maier, Jan-Steffen Pooth, Michelle Costa Galbas, Manuela Schön, David Boll, Georg Trummer, Christoph Benk, Jörg Haberstroh, Heidi Ramona Cristina Schmitz, Katharina Förster, Natalie Hoppe, Martin Büchsel, Simon Reiss, Martin Czerny, Wolfgang Bothe, Sam Brixius

**Affiliations:** 1 Department of Cardiovascular Surgery, Faculty of Medicine, University Heart Center Freiburg-Bad Krozingen, University of Freiburg, Hugstetter Strasse 55, 79106 Freiburg, Germany; 2 Center for Experimental Models and Transgenic Service, Medical Center, Faculty of Medicine, University of Freiburg, Stefan-Meier-Strasse 17, 79104 Freiburg, Germany; 3 Department of Cardiology and Angiology, Faculty of Medicine, University Heart Center Freiburg-Bad Krozingen, University of Freiburg, Hugstetter Strasse 55, 79106 Freiburg, Germany; 4 Department of Emergency Medicine, Medical Center, Faculty of Medicine, University of Freiburg, Hugstetter Strasse 55, 79106 Freiburg, Germany; 5 Institute of Clinical Chemistry and Laboratory Medicine, Medical Center, Faculty of Medicine, University of Freiburg, Hugstetter Strasse 55, 79106 Freiburg, Germany; 6 Department of Diagnostic and Interventional Radiology, Medical Center, Faculty of Medicine, University of Freiburg, Hugstetter Strasse 55, 79106 Freiburg, Germany

**Keywords:** Preclinical research, Diagnostic markers

## Abstract

Juvenile crossbred pigs are widely used for acute and chronic animal testing due to their anatomical and physiological resemblance to humans. They are particularly prevalent in preclinical cardiovascular research, including studies investigating extracorporeal resuscitation and mechanical circulatory support devices. However, the availability of comprehensive laboratory reference values is limited. In a single-center study at the University Medical Center Freiburg, Germany, the hematologic and biochemical laboratory values of anesthetized healthy juvenile German crossbred farm pigs were determined. Blood samples were collected at the beginning of surgical procedures, either arterially or venously. Females and males were compared, and correlation with body weight was assessed. In total, 268 animals (weight 57.8 ± 12.4 kg) were included, thereof 180 castrated males (55.2 ± 7.7 kg) and 79 females (63.6 ± 18.3 kg). There were significant differences between males and females in 11 of 45 parameters and a moderate correlation between body weight and creatinine (*R* = 0.41, *p* < 0.001). The reference intervals and insights into sex and body weight correlations enhance the utility of healthy juvenile German crossbred farm pigs in translational research, providing a robust reference for future studies.

## Introduction

Juvenile German crossbred farm pigs are commonly applied as large animal models for biomedical research and translational medicine in cardiovascular studies, such as investigations on extracorporeal resuscitation^1,2^, coronary interventions^3^, and left ventricular assist device implantation^4,5^. In general, pigs are frequently used as large animal models due to their anatomical and physiological similarities compared to humans^6^. Different breeds are used depending on the research aims and study duration. The most commonly applied breed in cardiovascular research is the crossbred farm pig (*Sus scrofa domesticus*)^7^. Knowing the reference intervals for laboratory parameters is mandatory for planning, conducting, interpreting, and optimizing animal studies with crossbred pigs. The primary purpose of this study was to provide reference intervals for hematological and biochemical parameters in anesthetized healthy juvenile domestic crossbred farm pigs. Moreover, we aimed to compare females and males and assess the correlation with body weight.

## Methods

### Animal study

In a single-center study at the University Medical Center Freiburg, Germany, the laboratory values of anesthetized healthy juvenile German crossbred farm pigs were determined between January 2011 and April 2024. The blood samples were routinely collected at the beginning of surgical procedures as part of multiple large animal studies. All experiments were approved by the local animal welfare committee (Regierungspräsidium Freiburg, Germany, G-08/39, G-10/90, G-15/148, G-19/169, G21/109, G-21/008, G-22/006, G-23/012, G-24/005). The research was conducted in accordance with the directive 2010/63/EU of the European Parliament and the Council^8^ and the ARRIVE guidelines^9,10^.

All experiments were performed on growing healthy juvenile German domestic crossbred farm pigs (German Landrace × Large White or [German Landrace × Large White] × Piétrain) aged approximately three to six months. The exact age of the animals was not documented. Following the German Pig Farming Hygiene Regulation, two local farmers in the southwest of Baden-Württemberg in southern Germany raised the animals under defined hygienic conditions^11^. Permission was obtained from the farmers to use their pigs in experiments. After animal purchase and delivery to the experimental animal facility of the University of Freiburg, the animals’ clinical condition was initially assessed by experienced staff veterinarians. Special attention was paid to transport-related injuries, signs of diarrhea (e.g., inspection of the anal area), breathing, and the general behavior of the animals while standing still and moving. The skin was checked for signs of ectoparasites (e.g., pig lice, mange) and bacterial infections (e.g., erysipelas). The pigs were fed a standard pellet chow (20 g/kg) and had access to water *ad libitum*. All animals were kept under controlled environmental conditions at 20 °C, 75 ± 5% humidity, and a 13/11-h light/dark cycle. If necessary, the pigs received iron supplementation as iron(III) oxide-hydroxide (200 mg/2 mL IV) two to three weeks before animal testing. After acclimatization and before an experiment, all animals were weighed and examined physically again, including their overall appearance, body condition, and behavior, for signs of illness or distress. Before surgery, the animals had no access to food for 12 hours. For premedication and sedation, the animals received ketamine (20 mg/kg IM) and midazolam (0.5 mg/kg IM). After preoxygenation, anesthesia was induced with propofol (2‒4 mg/kg IV). Following a bolus of vecuronium (0.2‒0.4 mg/kg/h IV) and endotracheal intubation with an endotracheal tube (size 7–8 mm), controlled mechanical ventilation was started and adjusted to maintain physiological conditions. In some animals (number unknown), potassium chloride (5 mL 7.45% IV) and magnesium chloride (1 g IV) were administered at the beginning of the surgery. Anesthesia was maintained with propofol (8‒15 mg/kg/h IV) or a mixture of isoflurane (1‒3%) and oxygen/air (FiO_2_ 30‒40%), and fentanyl (1–5 µg/kg/h). Vecuronium (0.2‒0.4 mg/kg/h IV) or cisatracurium (0.5‒0.7 mg/kg/h IV) were administered continuously for muscle relaxation. A full electrolyte solution (5‒10 mL/kg IV) was administered for fluid substitution. At the end of the experiments, while the animals were under deep general anesthesia, euthanasia was performed by intravenous injection of a potassium chloride solution (2 mmol/kg IV).

### Blood sampling and storage

Blood samples were collected from anesthetized animals at the beginning of surgical procedures either arterially (e.g., femoral artery) or venously (e.g., jugular vein, femoral vein) from an arterial or venous catheter (e.g., arterial line, central venous catheter). Usually, the following test tubes were used for sampling: 5.0 mL EDTA tubes (Sarstedt, Nümbrecht, Germany), 7.5 mL serum-separating tubes (Sarstedt), and 3.0 mL citrate tubes (Sarstedt). The preanalytical conditions were adopted from human samples. EDTA samples were measured within six hours. Citrated plasma and serum samples were spun immediately after arrival in the laboratory. If serum or plasma could not be measured within eight hours, they were kept at -20 °C and measured as a batch within one week.

In most cases, a blood gas analysis was also carried out. Since controlled mechanical ventilation was adjusted to maintain physiological conditions, the informative value of these results is limited. The results were not routinely stored and are not reported in this study.

### Blood analysis

The samples were analyzed by the Institute of Clinical Chemistry and Laboratory Medicine at the Medical Center of the University of Freiburg according to human standards. Hematologic parameters were measured by flow cytometry on an XN-9100 analyzer (Sysmex Deutschland GmbH, Germany). Blood smears were not reviewed. Serological parameters were measured by clinical chemistry or immunology on a Cobas 8000 analyzer (Roche, Switzerland) using reagents from Roche. Potassium, sodium, and chloride were measured with an ion-selective electrode. Coagulation assays were performed on a CS-5100 analyzer (Sysmex Deutschland GmbH, Germany) using reagents from Siemens Healthineers (Erlangen), except TAT, which was assessed by ELISA. Free hemoglobin was measured by photometry. When analyzers were exchanged during the study period, method comparisons were performed. Maintaining consistency in manufacturers and used reagents, no relevant changes in the reference ranges were noticed for the parameters evaluated in this study. For example, hematologic parameters were measured on an XN-9100 analyzer (Sysmex Deutschland GmbH, Germany) since 2021 and beforehand on XN-9000 analyzers. All instruments used were primarily validated for human samples in routine clinical use.

### Statistical analysis

The data was tested for normal distribution using the Shapiro-Wilk test and assessed graphically by histograms and quantile-quantile (QQ) plots. Normally distributed metric variables are reported as means with standard deviation (SD). For non-normally distributed variables, the medians and interquartile ranges are reported. Depending on sample size and data distribution, the lower and upper limits of the 95% reference intervals and the limits’ 90% confidence intervals were calculated. All calculations were performed in accordance with the *Guidelines for the Determination of Reference Intervals in Veterinary Species and other related topics* from the ASVCP Quality Assurance and Laboratory Standards Committee (QALS)^12^. When < 20 samples were available, the minimum and maximum were reported instead of the reference interval. Females and males were compared by mean differences (MD) and tested using a two-sided Student’s or Welch’s *t*-test. Pearson’s correlation coefficient was calculated to assess the correlation between laboratory values and body weight for samples with at least 30 data pairs. A *p*-value of < 0.05 was considered statistically significant. All calculations were performed using *R Statistical Software* (version 2021.9.0.351)^13^. Calculations of the lower and upper limits of the 95% reference intervals and their 90% confidence intervals were conducted using the *referenceIntervals* package (version 1.3.1)^14^. Data visualization was performed using the *ggplot2* package (version 3.4.2)^15^.

## Results

**Fig. 1:**
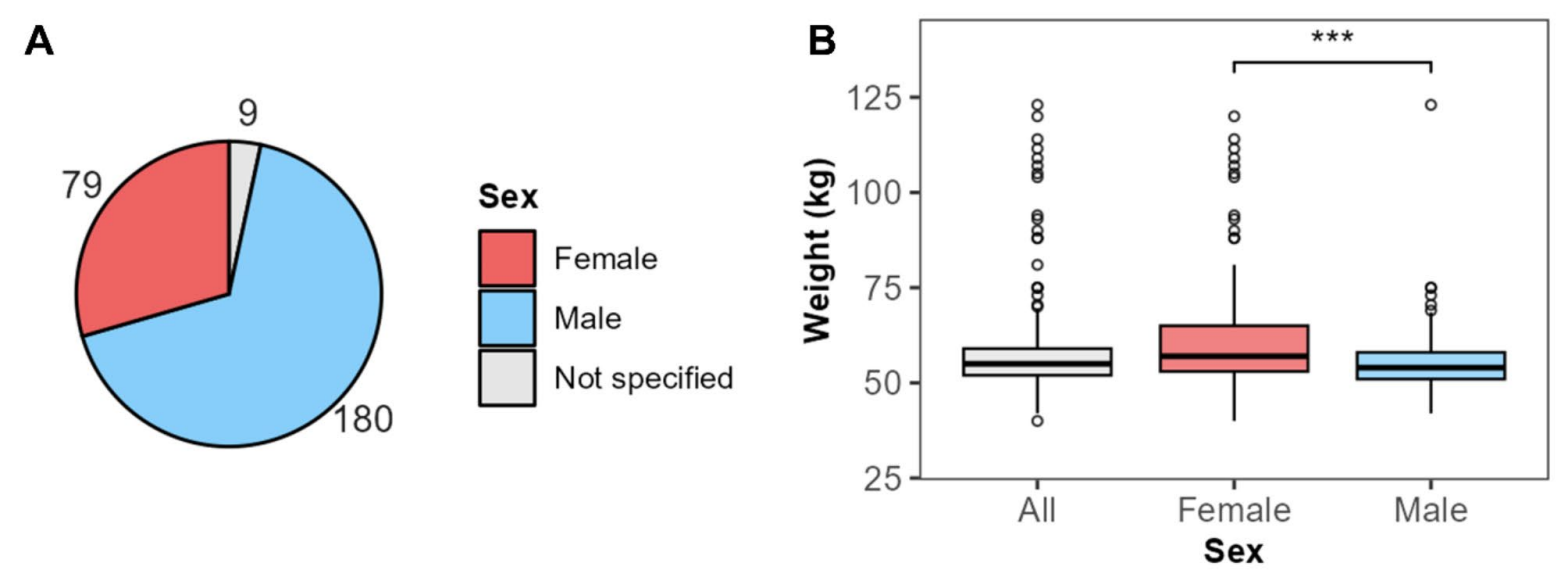
Animal characteristics. (**A**) Distribution of sex. (**B**) Body weight of all animals, females, and males. *** *p* < 0.001.

In total, 268 animals (79 female, 180 male, nine not specified) were included in the study. The mean weight was 57.8 ± 12.4 kg (females 63.6 ± 18.3 kg vs. males 55.2 ± 7.7 kg, *MD* = 8.4 kg, *p* < 0.001) (Fig. 1).

### Laboratory values

**Fig. 2:**
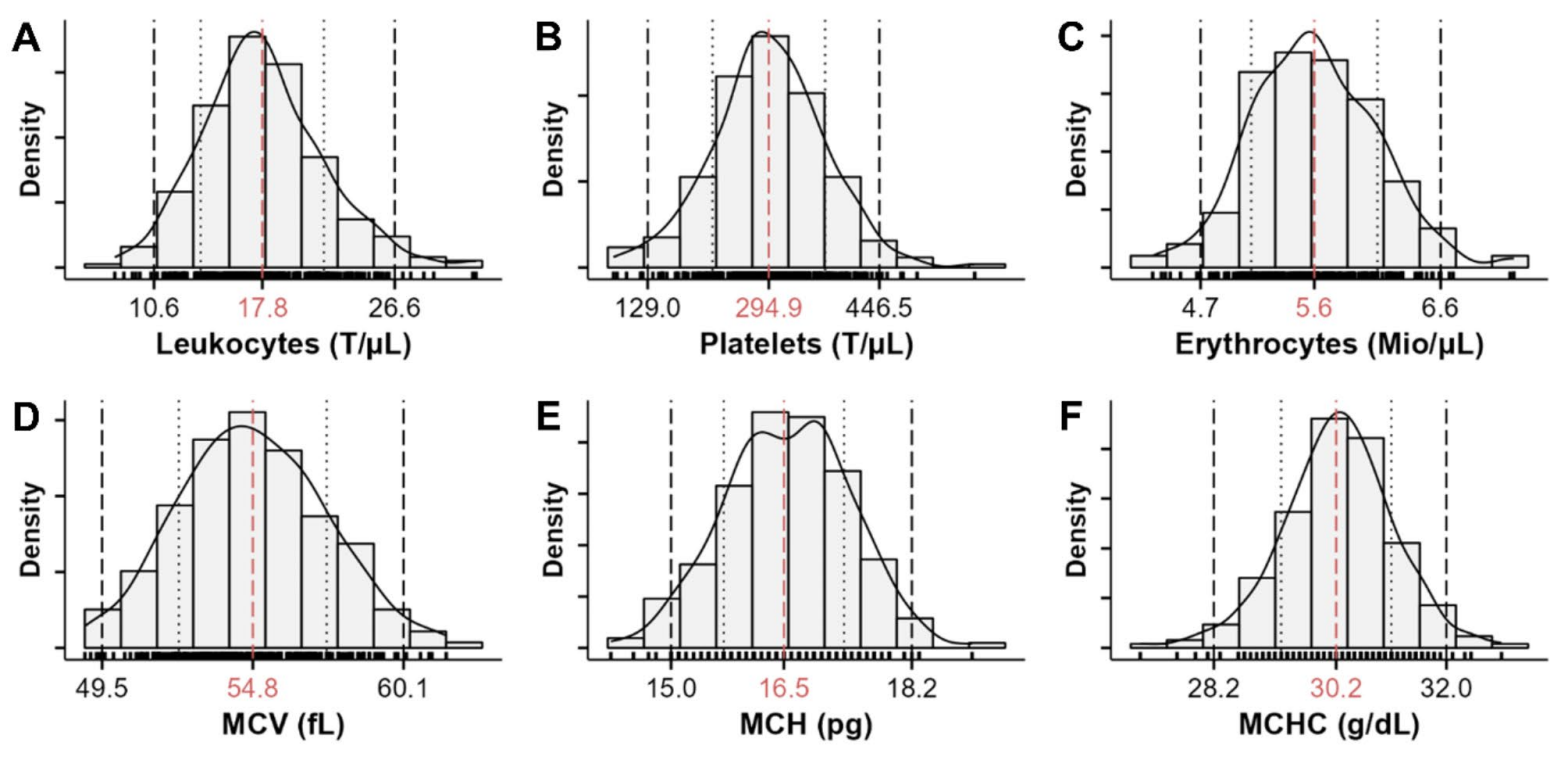
Density of selected hematological parameters. The dashed red line and the red number indicate the mean. The two dashed black lines, and the black numbers mark the lower and upper limits of the reference interval. The dotted grey lines represent the standard deviation. (**A**) Leukocyte count. (**B**) Platelet count. (**C**) Erythrocyte count. (**D**) Mean corpuscular volume (MCV). (**E**) Mean corpuscular hemoglobin (MCH). (**F**) Mean corpuscular hemoglobin concentration (MCHC).

The hematologic laboratory values are summarized in Table 1 and Fig. 2. The sample sizes used for the calculations were heterogeneous. For improved interpretation, the number of animals included is therefore also given. All primary blood cell counts were normally distributed. Monocyte and basophil counts were not normally distributed. Compared to human controls, the laboratory test reference intervals from the American Board of Internal Medicine (January 2024)^16^, pigs revealed an increased reference interval of the white blood cell count and lower values for hemoglobin and hematocrit, mean corpuscular volume, and hemoglobin.

**Table 1:** Hematologic parameters.

Variable	Unit	Pigs			Humans
		*N*	Mean ± SD	Lower limit [90% CI]–Upper limit [90% CI]	Lower–Upper
Leukocytes	T/µL	262	17.8 ± 4.1	10.6 [9.2–11.6]–26.6 [25.5–29.2]	4.0–11.0
Platelets	T/µL	259	294.9 ± 77.2	129.0 [100–155]–446.5 [423–479]	150–450
Erythrocytes	Mio/µL	259	5.6 ± 0.5	4.7 [4.4–4.8]–6.6 [6.5–7.2]	4.2–5.9
Hemoglobin	g/dL	259	9.3 ± 0.7	8.0 [7.8–8.2]–10.9 [10.7–11.0]	12–18
Hematocrit	%	259	30.7 ± 2.5	26.3 [25.1–27.1]–35.9 [35.2–38.0]	37–50
MCV	fL	218	54.8 ± 2.6	49.5 [49.1–50.7]–60.1 [59.2–61.1]	80–98
MCH	pg	218	16.5 ± 0.8	15.0 [14.5–15.1]–18.2 [17.9–18.3]	28–32
MCHC	g/dL	217	30.2 ± 0.9	28.2 [27.6–28.7]–32.0 [31.7–32.4]	33–36
RDW*	%	21	17.7 ± 1.6	15.9 [15.8–15.9]–22.5 [22.5–22.5]	9.0–14.5
Neutrophils	%	28	38.1 ± 12.4	13.8 [7.2–20.4]–62.3 [55.7–68.9]	50–70
Lymphocytes	%	28	57.8 ± 11.9	34.5 [28.2–40.8]–81.1 [74.8–87.4]	30–45
Monocytes*	%	28	3.0 ± 1.4	1.5 [1.3–1.5]–6.3 [6.3–7.1]	–6.0
Eosinophils	%	26	0.62 ± 0.40	0.1 [0.1–0.1]–0.8 [0.8–0.9]	–3.0
Basophils*	%	28	0.3 ± 0.2	0.0–0.8 [0.8–0.9]	–1.0
Neutrophils	T/µL	28	5.37 ± 2.17	1.12 [–2.27]–9.63 [8.48–10.79]	2.0–8.3
Lymphocytes	T/µL	28	8.15 ± 2.40	3.44 [2.17–4.72]–12.86 [11.58–14.13]	1.1–3.6^†^
Monocytes*	T/µL	28	0.43 ± 0.25	0.18 [0.14–0.18]–1.14 [1.14–1.46]	0.25–0.87^†^
Eosinophils	T/µL	22	0.10 ± 0.06	0.0–0.21 [0.18–0.25]	0.03–0.44^†^
Basophils*	T/µL	28	0.04 ± 0.03	0.1 [0.1–0.1]–0.14 [0.14–0.16]	0.01–0.08^†^

*MCH* mean corpuscular hemoglobin, *MCHC* mean corpuscular hemoglobin concentration, *MCV* mean corpuscular volume, *RDW* red blood cell distribution width, *SD* standard deviation.

*Non-normally distributed data, medians and interquartile ranges in Table 4.

^†^Human reference values from the Institute of Clinical Chemistry and Laboratory Medicine at the Medical Center of the University of Freiburg.

Table 2 shows the coagulation and plasma parameters. The porcine Quick, the international normalized ratio, von Willebrand factor antigen, and plasma-free hemoglobin were non-normally distributed.

**Table 2:** Coagulation and plasma parameters.

Variable	Unit	Pigs			Humans
		*N*	Mean ± SD	Lower limit [90% CI]–Upper limit [90% CI]	Lower–Upper
Quick*	%	186	91.4 ± 20.5	18 [10.4–44.0]–116 [112–126]	70–130^†^
INR*		184	1.11 ± 0.38	0.93 [0.87–0.94]–2.64 [1.51–3.72]	0.85–1.15^†^
PTT	sec	23	53.8 ± 15.7	23.1 [13.9–32.3]–84.5 [75.3–93.7]	25–35
TAT	µg/L	8	39.6 ± 21.3	10–82^§^	–4.3 ^22^
Fibrinogen	mg/dL	13	357 ± 28	312–403^§^	200–400
AT	%	184	90 ± 9	71.0 [65–75]–108.4 [106–115]	80–120
D-dimer	mg/L	12	0.40 ± 0.14	0.20–0.62^§^	–0.5
vWF antigen^*^	%	15	82 ± 35	53–184^§^	50–150
vWF activity	%	15	108 ± 19	75–143^§^	50–150
vWF ratio		15	1.76 ± 0.28	1.25–2.36^§^	0.7–1.1 ^23^
pfHb*	mg/dL	23	5.2 ± 4.1	0.2 [0.0–0.2]–15.0 [15–19]	–15.2 ^22^

*AT* antithrombin activity, *INR* international normalized ratio, *pfHb* plasma free hemoglobin, *PTT* partial thromboplastin time, *SD* standard deviation, *TAT* thrombin-antithrombin, *vWF* von Willebrand factor.

*Non-normally distributed data, median and interquartile range in Table 4.

^§^No calculation of reference interval due to sample size < 20, reporting of minimum and maximum.

^†^Human reference value from the Institute of Clinical Chemistry and Laboratory Medicine at the Medical Center of the University of Freiburg.

**Fig. 3:**
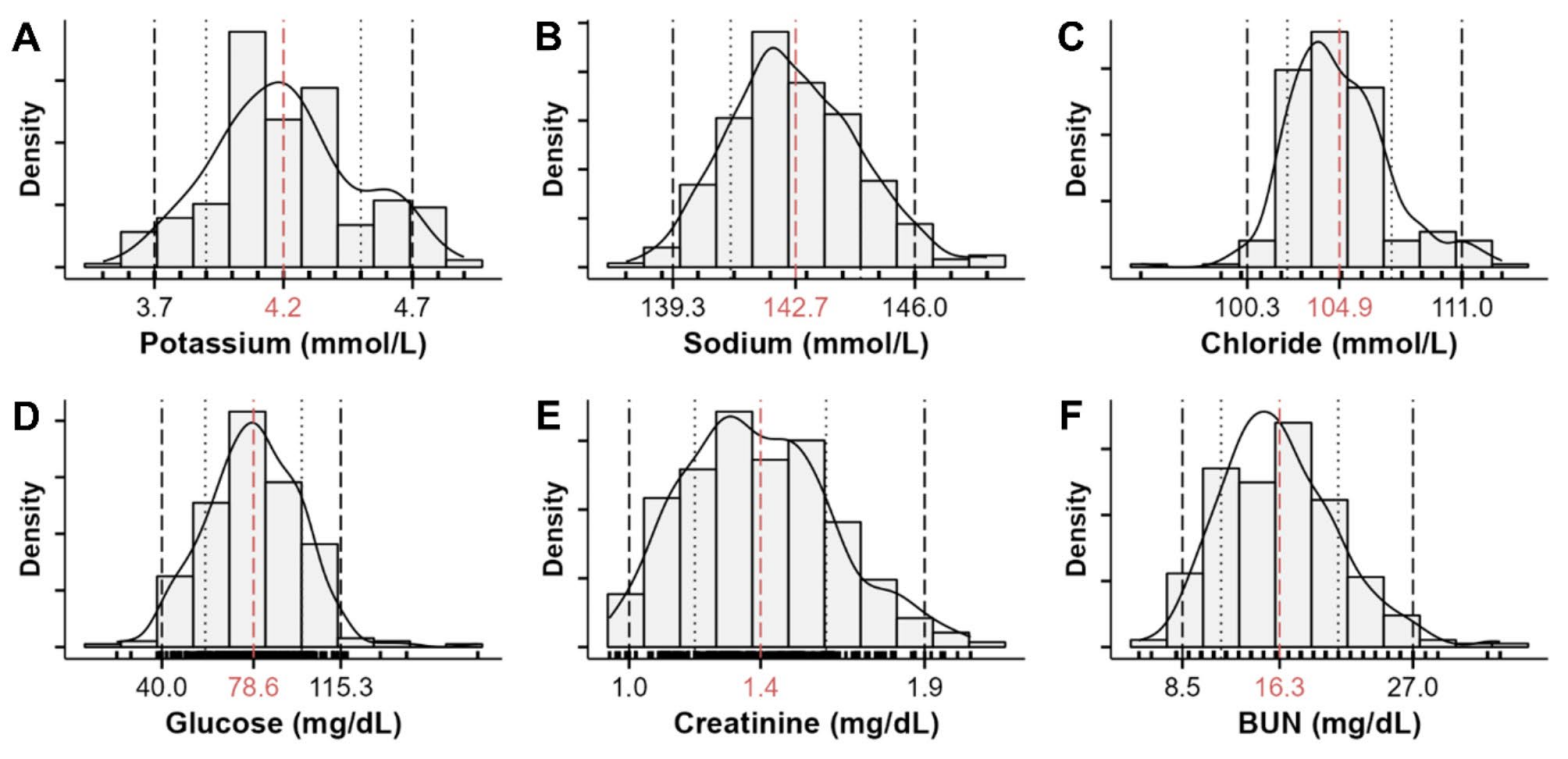
Density of selected biochemical serum parameters. The dashed red line and the red number indicate the mean. The two dashed black lines, and the black numbers mark the lower and upper limits of the reference intervals. The dotted grey lines represent the standard deviation. (**A**) Potassium. (**B**) Sodium. (**C**) Chloride. (**D**) Glucose. (**E**) Creatinine. (**F**) Blood urea nitrogen (BUN).

Serology is summarized in Table 3 and depicted in Fig. 3. Cystatin C, total and direct bilirubin, total creatine kinase, and ferritin were non-normally distributed. Compared to humans, pigs revealed higher reference intervals for creatinine and lactate dehydrogenase but lower values for certain electrolytes such as phosphorus, calcium, and magnesium, as well as haptoglobin.

**Table 3:** Serological parameters.

Variable	Unit	Pigs			Humans
		*N*	Mean ± SD	Lower limit [90% CI]–Upper limit [90% CI]	Lower–Upper
Potassium	mmol/L	253	4.2 ± 0.3	3.7 [3.6–3.8]–4.7 [4.7–4.8]	3.5–5.0
Sodium	mmol/L	249	142.7 ± 1.8	139.3 [139–140]–146.0 [146–148]	136–145
Chloride*	mmol/L	250	104.9 ± 2.6	100.3 [100–102]–111.0 [110–112]	98–106
Phosphorus	mmol/L	11	2.13 ± 0.17	1.87–2.42^§^	3.0–4.5
Calcium	mmol/L	251	2.50 ± 0.10	2.28 [2.22–2.32]–2.70 [2.69–2.72]	8.6–10.2
Magnesium	mmol/L	248	0.84 ± 0.10	0.66 [0.62–0.68]–1.06 [1.02–1.11]	1.6–2.6
Glucose	mg/dL	253	78.6 ± 20.3	40.0 [38–45]–115.3 [112–132]	70–99
Osmolality	mosm/kg	256	291 ± 6	282 [279–283]–303 [301–308]	275–295
BUN	mg/dL	260	16.3 ± 4.7	8.5 [8–9]–27.0 [25–29]	8–20
Creatinine	mg/dL	260	1.39 ± 0.22	1.00 [0.97–1.06] –1.88 [1.81–1.96]	0.50–1.3
Cystatin C*	mg/L	10	0.33 ± 0.09	0.22–0.47^§^	0.61–0.95^†^
Total bilirubin*	mg/dL	188	0.14 ± 0.11	0.08 [0.08–0.10]–0.30 [0.21–0.30]	0.3–1.0
Direct bilirubin*	mg/dL	8	0.11 ± 0.02	0.10–0.15^§^	0.1–0.3
AST, SGOT	U/L	260	26.7 ± 7.3	15.0 [13–16]–45.0 [41–52]	10–40
ALT, SGPT	U/L	260	42.1 ± 11.0	26 [25–28]–69 [66–73]	10–40
AP	U/L	11	109 ± 20	84–144^§^	30–120
GGT	U/L	11	31.3 ± 5.7	22–43^§^	8–50
Total protein	g/dL	15	5.02 ± 0.24	4.5–5.4^§^	5.5–9.0
Albumin	g/dL	14	3.43 ± 0.22	3.0–3.8^§^	3.5–5.5
LDH	U/L	23	478 ± 70	341 [300–382]–614 [573–655]	80–225
Total CK*	U/L	254	2341 ± 2090	730 [565–862]–9325 [6740–11203]	30–170
CK-MB	U/L	254	279 ± 91	138 [127–144]–524 [463–562]	0–24^†^
Myoglobin	ng/mL	14	30.4 ± 9.1	17–50^§^	0.0–100
Haptoglobin	mg/dL	9	11.6 ± 4.2	6–21^§^	83–267
NSE	µg/L	234	0.6 ± 0.2	0.2 [0.2–0.3]–1.1 [0.9–1.1]	–17^†^
Ferritin*	ng/mL	98	25.1 ± 37.3	5 [5–5]–169 [148–217]	24–336

*ALT* alanine aminotransferase, *AP* alkaline phosphatase, *AST* aspartate aminotransferase, *BUN* blood urea nitrogen, *CK* creatine kinase, *CK-MB* creatine kinase muscle-brain type, *LDH* lactate dehydrogenase, *NSE* neuron-specific enolase, *SD* standard deviation, *SGOT* serum glutamic oxaloacetic transaminase, *SGPT* serum glutamic pyruvate transaminase.

*Non-normally distributed data, median and interquartile range in Table 4.

^§^No calculation of reference interval due to sample size < 20, reporting of minimum and maximum.

^†^Human reference value from Institute of Clinical Chemistry and Laboratory Medicine at the Medical Center of the University of Freiburg.

For non-normally distributed variables, the median and interquartile range are given in Table 4.

**Table 4:** Medians and interquartile ranges of non-normally distributed variables.

Variable	Unit	*N*	Pigs	
			Median	IQR
Hematology				
RDW	%	21	17.6	16.5–18.5
Monocytes	%	28	2.40	2.05–3.70
Basophils	%	28	0.20	0.18–0.33
Monocytes	T/µL	28	0.37	0.26–0.44
Basophils	T/µL	28	0.03	0.02–0.05
Coagulation and plasma				
Quick		184	96	86–104
INR	sec	23	1.03	0.99–1.07
vWF antigen	%	15	69	66–76
pfHb	mg/dL	23	3.2	2.0–8.3
Serum				
Chloride	mmol/L	250	105	103–106
Cystatin C	mg/L	10	0.28	0.26–0.42
Direct bilirubin	mg/dL	8	0.11	0.11–0.12
Total bilirubin	mg/dL	187	0.10	0.10–0.20
Total CK	U/L	254	1786	1357–2484
Ferritin	ng/mL	98	12.0	7.0–24.8

*CK* creatine kinase, *IQR* interquartile range, *pfHb* plasma free hemoglobin, *RDW* red blood cell distribution width, *vWF* von Willebrand factor.

### Differences between males and females

There were significant differences between males and females in eleven parameters (Table 5). Males revealed a greater number of leukocytes (*p* = 0.013) and erythrocytes (*p* < 0.001), more hemoglobin (*p* = 0.013) but slightly smaller mean corpuscular volume (*p* < 0.001) and mean corpuscular hemoglobin (*p* = 0.003). Females showed a shorter partial thromboplastin time (*p* = 0.005), lower antithrombin activity (*p* = 0.048), and higher plasma-free hemoglobin concentration (*p* < 0.001). Males had a lower creatinine (*p* = 0.030) but greater lactate dehydrogenase (*p* = 0.002) and creatine kinase (muscle-brain type) concentration (*p* = 0.004).

**Table 5:** Significant differences between males and females.

Variable	Unit	Female		Male		MD	*p*
		*N*	Mean ± SD	*N*	Mean ± SD		
Hematology							
Leukocytes	T/µL	77	16.8 ± 4.0	178	18.2 ± 4.1	1.4	0.013
Erythrocytes	Mio/µL	76	5.42 ± 0.47	176	5.66 ± 0.48	0.24	< 0.001
Hemoglobin	g/dL	76	9.1 ± 0.7	176	9.3 ± 0.7	0.2	0.013
MCV	fL	63	55.7 ± 2.2	150	54.4 ± 2.6	1.3	< 0.001
MCH	pg	63	16.8 ± 0.8	150	16.4 ± 0.8	0.4	0.003
Coagulation and plasma							
PTT	sec	49	70.4 ± 26.3	109	83.4 ± 24.5	12.9	0.005
AT	%	51	87.9 ± 8.0	129	90.6 ± 9.1	2.7	0.048
pfHb	mg/dL	13	7.6 ± 4.1	10	2.1 ± 0.9	5.5	< 0.001
Serum							
Creatinine	mg/dL	76	1.43 ± 0.22	177	1.37 ± 0.23	0.07	0.030
LDH	U/L	13	442 ± 58	10	525 ± 55	83	0.002
CK-MB	U/L	74	254 ± 91	174	291 ± 89	37	0.004

*AT* antithrombin activity, *CK-MB* creatine kinase muscle-brain type, *LDH* lactate dehydrogenase, *MCH* mean corpuscular hemoglobin, *MD* mean difference, *MCV* mean corpuscular volume, *pfHb* plasma free hemoglobin, *PTT* partial thromboplastin time, *SD* standard deviation.

### Correlation with body weight

**Fig. 4:**
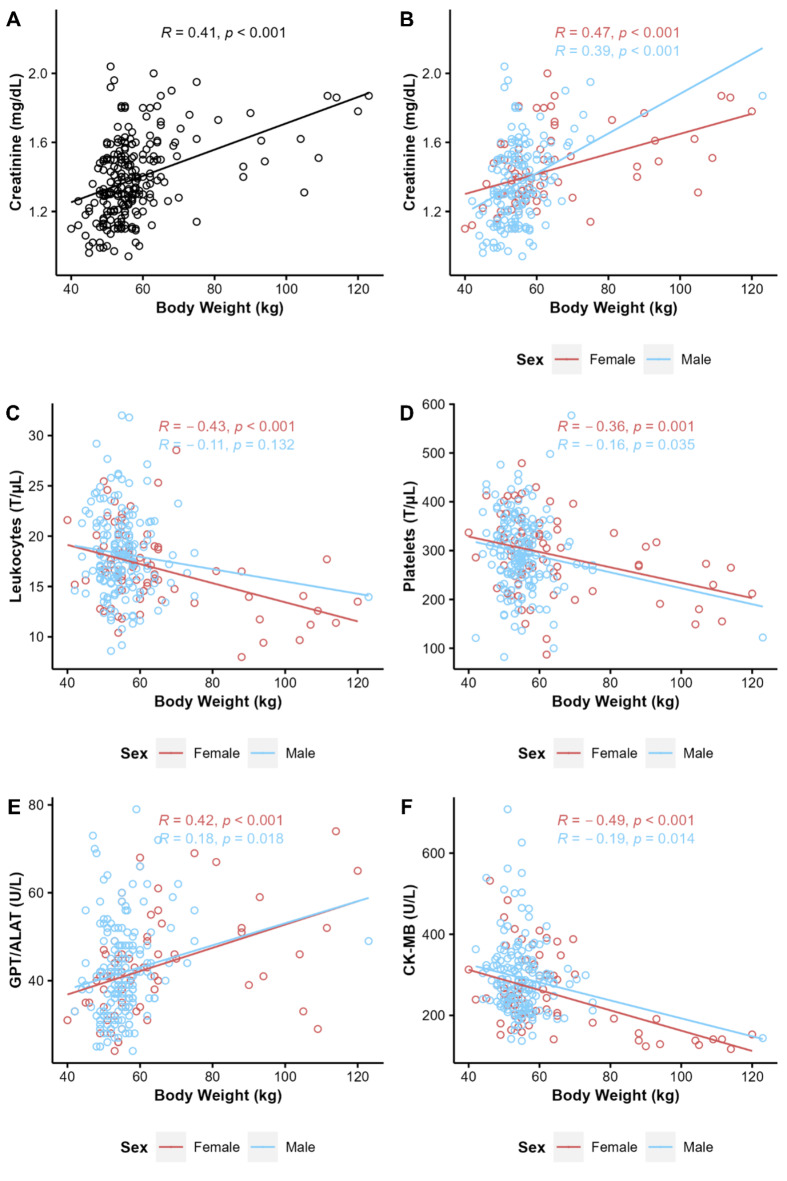
Correlation between body weight and different parameters. (**A**) Creatinine. Dependent on sex: (**B**) creatinine. (**C**) Number of leukocytes and (**D**) platelets. (**E**) Serum glutamic pyruvate transaminase (GPT) or alanine aminotransferase (ALAT). (**F**) Creatine kinase muscle-brain type (CK-MB).

Assessing sample sizes greater than or equal to 30 for correlation with body weight, there was only a moderate correlation with creatinine (*R* = 0.41, *p* < 0.001). A subgroup analysis by sex revealed that females showed an additional negative correlation between body weight and the number of leukocytes (*R* = − 0.43, *p* < 0.001) and platelets (*R* = − 0.36, *p* = 0.001) as well as creatine kinase muscle-brain type (*R* = − 0.49, *p* < 0.001) (Fig. 4).

## Discussion

The present study provides comprehensive reference values for hematological and biochemical parameters in anesthetized healthy juvenile German domestic crossbred farm pigs, highlighting significant differences between sexes and correlations with body weight. Applying pigs as animal models for biomedical research and translational medicine, studying, for example, cardiovascular diseases such as heart failure^5^, extracorporeal resuscitation^1^, and coronary artery disease^3^, it is crucial to have accurate laboratory reference values. It aids in accurately interpreting test results, enabling researchers to distinguish between normal variations and deviations indicative of disease or experimental conditions^17^. It contributes to the refinement of animal experiments by better understanding the physiological state and potential sources of variation in the animal model. Reference intervals specific to age groups, such as juvenile pigs, are necessary because adult reference intervals may not be applicable due to age-related changes. By having age-appropriate and breed-specific reference intervals, scientists can more accurately interpret laboratory data, enhance the validity of their findings, and ultimately improve the translation of results to human medicine^17,18^.

The main findings of this study are as follows. Over three-quarters of all investigated variables exhibited a normal distribution pattern, indicating high conformity to expected parametric assumptions. Remarkably, the observed values demonstrated remarkable similarity to established human reference intervals, underscoring the translational relevance of the study model. However, a significant difference emerged between males and females, highlighting the importance of considering sex-specific variations in data interpretation. The correlation between laboratory values and body weight was poor, suggesting that these parameters may not be strongly influenced by variations in body mass within the study population.

Porcine laboratory values can vary significantly depending on intrinsic and extrinsic factors related to the animals and their environment. For instance, age is a crucial determinant, as newborns and young piglets have markedly different hematological and biochemical parameters than adults^17,18^. There are also significant differences between nursery pigs and sows^19^. Contrary to our study, Li et al. found no differences between male and female Landrace pigs^20^. Furthermore, intramuscular anesthesia induction prior to blood sampling can affect parameters associated with muscular injury. In our study, for example, creatine kinase levels were significantly elevated compared to human reference values, likely due to the intramuscular induction of anesthesia. Environmental stressors, such as extreme temperatures and exposure to pathogens, can also alter porcine blood parameters^21^.

This study has several limitations. First, it can only inform on laboratory values from a single center. It focused solely on German crossbred farm pigs from two local distributors, limiting generalizability to other pig breeds. The research was restricted to a narrow weight range, which may not apply to pigs of different stages or sizes. The age of the animals was not particularly documented. The sample size for different parameters was heterogeneous, affecting the reliability of some results. In a few cases, extended storage times of blood samples potentially compromised the validity and accuracy of subsequent laboratory tests. Future studies should include diverse breeds, ages, and weights, maintain consistent lab practices, and minimize missing data to enhance validity.

Overall, these reference values and insights into sex and body weight correlations enhance the utility of healthy juvenile crossbred pigs in translational research, providing a robust baseline for future studies. They facilitate the accurate assessment of pathological changes and therapeutic interventions, thereby improving the reliability and reproducibility of preclinical findings. Further research should expand these reference intervals and explore additional parameters to fully characterize the physiological and biochemical landscape of juvenile pigs.

## Data Availability

The datasets used and analyzed during the current study are available from the corresponding author upon reasonable request.
